
JOURNAL INFORMATION
==============================
NLM Title Abbreviation: EMBO Mol Med
Journal ID: EMBO Mol Med
NLM Journal ID: 101487380
Journal ID: emmm

EMBO Molecular Medicine

ISSN: 1757-4676
EISSN: 1757-4684
Publisher: Nature Publishing Group

ARTICLE INFORMATION
==============================
PMCID: PMC3840481
DOI: 10.1002/emmm.201370111
Article version: 1
Subjects: Masthead

Masthead (EMBO Mol. Med. 11/2013)

Print publication date: 2013 Nov
Electronic publication date: 2013 Nov 4
Volume: 5
Issue: 11
First page: i
Last page: ii
Copyright: © 2013 The Authors. Published by John Wiley and Sons, Ltd on behalf of EMBO
Copyright year: 2013
License: Re-use of this article is permitted in accordance with the Creative Commons Deed, Attribution 2.5, which does not permit commercial exploitation.
License URL: https://creativecommons.org/licenses/by/2.5/

==============================
EMBO
Molecular Medicine

### Advisory Board

**Adriano Aguzzi**, Zürich
**Kari Alitalo**, Helsinki
**Russ B. Altman**, Stanford
**Stylianos Antonarakis**, Geneva
**Alan Ashworth**, London
**Karen B. Avraham**, Tel Aviv
**Andrea Ballabio**, Napoli
**Yann Barrandon**, Lausanne
**Anton J. Berns**, Amsterdam
**Clara D. Bloomfield**, Columbus
**Pico Caroni**, Basel
**Vivian Cheung**, Philadelphia
**Kenneth R. Chien**, Massachusetts
**Hans Clevers**, Utrecht
**Elisabetta Dejana**, Milano
**Peter Dirks**, Toronto
**Oliver Eickelberg**, Munich
**Martin Eilers**, Marburg
**Marc Feldmann**, London
**Alain Fischer**, Paris
**Philippe Froguel**, London
**Gerardo Gamba**, Mexico City
**Martin Gleave**, Vancouver
**Frank G. Grosveld**, Rotterdam
**Christian Haass**, München
**Carl-Henrik Heldin**, Uppsala
**Doug Hilton**, Melbourne
**Jan Hoeijmakers**, Rotterdam
**Gokhan Hotamisligil**, Boston
**Nancy E. Hynes**, Basel
**Alain Israel**, Paris
**Barbara B. Kahn**, Boston
**Cynthia Kenyon**, San Francisco
**Robert S. Kerbel**, Toronto
**Jean-Pierre Kinet**, Boston
**Jonathan K. C. Knowles**, Basel
**Andreas E. Kulozik**, Heidelberg
**Guido Kroemer**, Paris
**Christopher J. Marshall**, London
**Joan Massagué**, New York
**Anthony P. Monaco**, Medford
**Salvador Moncada**, London
**Pierluigi Nicotera**, Bonn
**Bernd Nilius**, Leuven
**Eric N. Olson**, Dallas
**Stephen O'Rahilly**, Cambridge
**Albert Osterhaus**, Rotterdam
**Manolis Pasparakis**, Köln
**Christine Petit**, Paris
**Kornelia Polyak**, Boston
**Rino Rappuoli**, Siena
**Peter J. Ratcliffe**, Oxford
**Gideon Rechavi**, Tel Aviv
**Paola Ricciardi-Castagnoli**, Singapore
**Nadia Rosenthal**, Monterotondo
**Richard Schilsky**, Chicago
**Nicholas J. Schork**, La Jolla
**Yosef Shiloh**, Ramat Aviv
**Gerald Shulman**, New Haven
**Austin G. Smith**, Cambridge
**Bruce Spiegelman**, Boston
**Deepak Srivastava**, San Francisco
**Giulio Superti-Furga**, Vienna
**Elaine I. Tuomanen**, Memphis
**Axel Ullrich**, Martinsried
**Veronica van Heyningen**, Edinburgh
**Inder M. Verma**, La Jolla
**Simon Wain-Hobson**, Paris
**Gerd Walz**, Freiburg
**Fiona Watt**, London
**Christian Weber**, München

### Senior Editors

**Dario Alessi**, Dundee
**Giulio Cossu**, London
**Uta Francke**, Stanford
**Fred Gage**, San Diego
**Matthias Hentze**, Heidelberg
**Edison T. Liu**, Bar Harbor
**Philippe Sansonetti**, Paris
**Bart de Strooper**, Leuven

### Chief Editor

**Stefanie Dimmeler**

### Editors

**Roberto Buccione**
**Céline Carret**

### Head of Scientific Publications

**Bernd Pulverer**

### Deputy Head of Scientific Publications

**Thomas Lemberger**

### Editorial Office

EMBO Molecular Medicine
Meyerhofstrasse 1
69117 Heidelberg
Germany
Tel: +49 6221 8891 310
Fax: +49 6221 8891 240
E-mail: info@embomolmed.org

### Publisher

Wiley-Blackwell
John Wiley & Sons, Ltd.
The Atrium,
Southern Gate, Chichester, West Sussex
PO19 8SQ, UK
E-mail: info@embomolmed.org

### Indexed in

MEDLINE/PubMed (NLM)
Science Citation Index Expanded
Journal Citation Reports/Science Edition
Biological Abstracts
BIOSIS Previews
SCOPUS (Elsevier)

www.embomolmed.org

EMBO excellence in life sciences

WILEY-BLACKWELL

EMBO Molecular Medicine

Open Access is published 12 times a year

Online ISSN 1757-4684

© 2013 EMBO
69117 Heidelberg, Germany

COPE COMMITTEE ON PUBLICATION ETHICS

*This journal is a member of and subscribes to the principles of the Committee on Publication Ethics*

### Aims and Scope

*EMBO Molecular Medicine* is a peer-reviewed, open access online journal dedicated to a new research discipline at the interface between clinical research and basic biology. It offers clinicians and researchers in this area the opportunity to publish their best work in a broadly distributed and highly visible forum, thereby lending a strong impetus to this important and rapidly developing field and helping to forge new links between clinicians and molecular biologists. Studies based on model organisms also fall within the scope of the journal, provided that the results presented are evidently and directly relevant to human disease.

*EMBO Molecular Medicine* publishes research articles as full-length research papers and short reports. In addition, the journal publishes editorials and review articles in innovative formats that target a broad and non-specialized audience. For further information visit www.embomolmed.org.

*EMBO Molecular Medicine* is now part of the Wiley Open Access publishing program of fully open access journals published by Wiley. For further information visit the Wiley Open Access website at http://www.wileyopenaccess.com.

### Open Access and Copyright

All articles published by *EMBO Molecular Medicine* are fully open access: immediately freely available to read, download and share. All *EMBO Molecular Medicine* articles accepted from 14 August 2012 are published under the terms of the Creative Commons Attribution 3.0 Unported (CC BY 3.0) license, which permits use, distribution and reproduction in any medium, provided the original work is properly cited. Articles accepted before this date were published under the agreement as stated in the final article.

Copyright on any *EMBO Molecular Medicine* article accepted after 7 March 2012, the date on which *EMBO Molecular Medicine* became an open access journal, is retained by the author(s). Authors grant to EMBO and Wiley a license to publish the article and for Wiley to identify itself as the original publisher. Authors also grant any third party the right to use the article freely as long as its integrity is maintained and its original authors, citation details and publisher are identified.

### Use by commercial "for-profit" organisations (not applicable to CC BY 3.0 licensed articles)

Use of Wiley Open Access articles for commercial, promotional, or marketing purposes requires further explicit permission from Wiley and will be subject to a fee. Requests to reproduce material from this journal content are being handled through the Rightslink® online service.

- Locate the article you wish to reproduce on Wiley Online Library (http://onlinelibrary.wiley.com)
- Navigate to the abstract page
- Click on the 'Request Permissions' link, under the 'ARTICLE TOOLS' menu
- Follow the online instructions and select your requirements from the drop down options and click on 'quick price' to get a quote
- Create a RightsLink® account to complete your transaction (and pay, where applicable)
- Read and accept our Terms & Conditions and Download your license
- For any technical queries please contact customercare@copyright.com
- For further information and to view a Rightslink® demo please visit www.wiley.com and select Rights & Permissions.

Special requests should be addressed to rightsDE@wiley.com. Print reprints of Wiley Open Access articles can be purchased from corporatesalesDE@wiley.com.

### Disclaimer

The Publisher and Editors cannot be held responsible for errors or any consequences arising from the use of information contained in this journal; the views and opinions expressed do not necessarily reflect those of the Publisher and Editors, neither does the publication of advertisements constitute any endorsements by the Publisher and Editors of the products advertised.

Wiley Open Access articles posted to repositories or websites are without warranty from Wiley of any kind, either express or implied, including, but not limited to, warranties of merchantability, fitness for a particular purpose, or non-infringement. To the fullest extent permitted by law Wiley disclaims all liability for any loss or damage arising out of, or in connection, with the use of or inability to use the content.
